# Reviewed article: Pimenta IT, Marsico EFC, Souto APM, Geraldo MCHM, Amaral LF, Duffrayer KM, Ribeiro CLP, Aguilar GMO. Vigilância em saúde em eventos de massa: relato da experiência do Centro de Informações Estratégicas em Vigilância em Saúde da cidade do Rio de Janeiro, 2024. Epidemiol Serv Saude. 2025:34;e20250212

**DOI:** 10.1590/S2237-96222025v34e20250212.a

**Published:** 2025-09-29

**Authors:** Jean Ezequiel Limongi

**Affiliations:** 1 Universidade Federal de Uberlândia, Uberlândia, MG, Brazil

## Questionnaire

Does the manuscript contain new and significant information to justify publication? Yes

Does the Abstract (Summary) clearly and accurately describe the content of the article? Yes

Is the problem significant and concisely stated? Yes

Are the methods described comprehensively? Yes

Are the interpretations and conclusions justified by the results? Yes

Is adequate reference made to other work in the field? Yes

## Manuscript Structure

Length of article is: Too short

Number of tables is: Adequate

Number of figures is: Adequate

Please state any conflict(s) of interest that you have in relation to the review of this paper. None

Interest: Excellent

Quality: Good

Originality: Excellent

Overall: Good

Recommendation: Major Revision

## Comments

O manuscrito é relevante e apresenta dados que não são frequentemente apresentados em revistas acadêmicas. Além disso, é bem escrito e organizado. Por isso, acredito que a seção de discussão mereça mais atenção e elaboração dos autores.

